# The battle over “food addiction”

**DOI:** 10.3389/fpsyt.2025.1621742

**Published:** 2025-08-28

**Authors:** Robert H. Lustig

**Affiliations:** ^1^ Department of Pediatrics, University of California San Francisco, San Francisco, CA, United States; ^2^ Institute for Health Policy Studies, University of California San Francisco, San Francisco, CA, United States

**Keywords:** food addiction, sugar, insulin, dopamine, leptin, food industry, ultraprocessed foods

## Abstract

Despite decades of nutrition, obesity, and diabetes research, and worsening prevalences and severities of virtually every chronic metabolic disease, the scientific community remains divided over the existence and veracity of the concept of food addiction. There are numerous rationalizations — 1) you need food to survive, (of which “Food is Medicine” is the latest mantra); 2) people with obesity should not be stigmatized as “mentally ill”; 3) people with obesity should instead adhere to “personal responsibility”; 4) the data are incomplete and not strong enough; 5) it’s correlation but not causation; 6) everyone is exposed, but not everyone is addicted; 7) there is no “withdrawal” phenotype; and 8) it’s not “food addiction” but “eating addiction”. All are in play, yet more health care dollars are diverted to the treatment of food-related disease every year. While various ingestible chemicals (e.g. nicotine, cocaine, heroin, alcohol) are clearly addictive, it appears to be a stretch by some scientists to argue that individual substances found in food (e.g. sugar, caffeine), or the food itself (e.g. ultraprocessed food), rise to meet the same criteria. Symposia on food addiction proliferate and journal debates continue. The definition of addiction consists of numerous criteria, including public health demographics, biochemistry, imaging, animal trials, clinical trials, and economics. None of these have proven to be “slam dunks” to align a general consensus. But paramount for scientific acceptance is the delineation of mechanism. This article will review the history of the controversy, the data on which foods are most likely to be addictive, the two mechanisms involved in the pathogenesis of food addiction and relate it to the most likely culprits, and the role of the food industry in promulgating false narratives, in order to provide a rational way forward from this debate.

## Introduction

Non-communicable diseases (NCD’s) now account for 72% of deaths (1) and 75% of health care dollars (2) in the United States. The seminal role of the Western Diet in these pandemics is unchallenged; even the beverage companies have capitulated (3). Every country that adopts the Western diet is burdened with these same diseases and resultant costs. The question now is “what to do about it?” The prerequisite question: is it the quantity or the quality of these foods that are to blame? This is not a semantic argument. Quantity is determined by the end user (a personal responsibility issue); while quality is determined by manufacturers (a public health issue). But what if the quality altered the quantity? Those that favored either view over the other would thus appear to be justified within their own stance. Clearly, hyperpalatable food is “hyperpalatable” that is, the majority of people can’t stop eating them. The question is why? Is it because they *like* to? Or because they *want* to? Or because they *need* to? This argument seems to have drawn to an academic stalemate over the concept of “food addiction” that certain foods act in the brain like drugs of abuse, in which the biochemical drive to consume is greater than the brain’s cognitive restraint to stop (4).

First, my personal disclosure — I do believe that certain substances found in food are addictive. I came to this conclusion slowly through my own research and from the science of others. From the discovery of leptin in 1994, I thought that the homeostatic pathway of energy balance was enough to explain obesity. However, by 2009 I realized that this theory was woefully inadequate. And since then, the science has been rolling in. I’m a member of the International Food Addiction Consensus Conference (IFACC) Working Group to get the World Health Organization and the American Psychiatric Association to adopt the concept of food addiction into their compendia of psychiatric disease. Therefore, I present this chapter cognizant of my somewhat biased mindset, having myself “drunk the Kool-Aid”. Nonetheless, I will endeavor to elaborate the arguments from the other side of this contentious debate.

As the research has been elaborated, it would appear that foods normally found in nature are not addictive; rather it is the purification and mixing of specific components and ingredients, and the removal of fiber, that renders some foods as addictive. This has led the field away from the concept of generalized “food addiction” and toward to a more refined concept of “ultraprocessed food (UPF) addiction”. Nevertheless, detractors continue to abound.

## History of the “food addiction” controversy

The first allusion to the concept of food addiction dates back to 1956, when Randolph casually introduced the concept while describing alcohol addiction (5). This should not be surprising, as the biochemical, hedonic and social similarities between alcohol and sugar are virtually identical (6). However, the concept of food addiction was not initially embraced by the psychiatric profession; and even today there remains a great deal of skepticism. For instance, the DSM-IV published in 1993 listed “substance use disorder” as requiring *both tolerance and withdrawal* as necessary criteria for the definition of addiction, yet (apart from caffeine and ethanol) no ingredient found in food demonstrated obvious withdrawal. However, as the public health difficulties stemming from addiction expanded, the definition, of necessity, expanded. Some investigators argued that specific components of processed food, and in particular those in “fast food”, are addictive in a manner similar to nicotine, alcohol, cocaine, and heroin (7). The DSM-5, published in 2013, reclassified the field of addiction to include “behavioral addictions” that did not have a chemical withdrawal paradigm (e.g. gambling, video games, social media, pornography). Thus, a revised set of criteria incorporating *psychological dependence* was proffered by the DSM-5 (8), which might be better fitted to the concept of food addiction, including:

1. Craving or a strong desire to use;
2. Recurrent use resulting in a failure to fulfill major role obligations (work, school, home);
3. Recurrent use in physically hazardous situations (e.g. driving);
4. Use despite social or interpersonal problems caused or exacerbated by use;
5. Taking the substance or engaging in the behavior in larger amounts or over a longer period than intended;
6. Attempts to quit or cut down;
7. Time spent seeking or recovering from use;
8. Interference with life activities;
9. Use despite negative consequences.

The next scientific salvo in this battle occurred in 2008, based on the work of Nicole Avena in Bart Hoebel’s lab at Princeton University which demonstrated that sugar satisfied the four criteria for addiction in animals: bingeing, withdrawal, craving, and cross-sensitization with other drugs of abuse (9). This led to a flurry of activity in 2009–2010 in the midst of the obesity epidemic, when U. Florida psychiatrist Mark Gold planted his flag in the ground with two seminal volumes addressing food addiction (10, 11). Instead of acceptance, the result was an academic backlash. A group led by Cambridge psychiatrist Paul Fletcher directly challenged the addiction model of obesity (12) based on the notion that food cannot be addictive as it is essential to survival. This led to a heated back-and-forth exchange between Nicole Avena and the Cambridge group, with no resolution (13, 14).

In 2009, Ashley Gearhardt working with Kelly Brownell at Yale, alluded to the addictiveness of the Western diet (15), driving excessive consumption. The Yale Food Addiction Scale (YFAS) logs specific foods as having addictive properties (16), and a children’s YFAS also reveals that food addiction is common, especially in obese youth (17). While there is general acceptance as to the phenomenon of tolerance to ultraprocessed food in humans, there is much more debate about the existence of withdrawal. There are anecdotal data of withdrawal (18), and a Highly Processed Food Withdrawal Scale (ProWS) has been developed for both adults (19) and children (20). While empiric evidence for sugar withdrawal in humans appears adequate (21), proving it remains a priority. To this end, our group at UCSF, using the opiate antagonist naltrexone as a probe for reward, discerned a phenomenon called “Reward Eating Drive” (RED), which belies those obese individuals who appear to respond excessively to hedonic food cues (22, 23). Furthermore, using functional MRI (fMRI) studies, other investigators have defined the prefrontal cortex as responsible for the response of sweet tastes as being “attractive” or “unattractive” (24). These data suggest that naltrexone interfered with endogenous opioid peptide (EOP) tone that mediated these cravings, going a long way to codify the concept of food addiction.

These epidemiologic and mechanistic findings have not placated the naysayers. A group of academics organized in Europe under the banner of “NeuroFAST”, which does not accept the concept of food addiction (25), instead calling the overconsumption of hyperpalatable food “eating addiction” (26) and it is the behavior, rather than the food, that distinguishes the phenomenon. This distinction is not semantic, because if it is food addiction, the food industry bears culpability, whereas if it is eating addiction, the consumer bears that culpability. The NeuroFAST investigators state that even though specific foods can generate a reward signal, they are not addicting, and those that do *are not considered as food*. In their own words:

“In humans, there is no evidence that a specific food, food ingredient or food additive causes a substance-based type of addiction (the only currently known exception is caffeine which via specific mechanisms can potentially be addictive). Within this context we specifically point out that we do not consider alcoholic beverages as food, despite the fact that one gram of ethanol has an energy density of 7 kcal (27)”.

NeuroFAST recognizes caffeine as addictive, yet it absolves it as a driver of food addiction. Xanthine alkaloids are present naturally in many foods, yet caffeine is classified by the FDA as a food additive. It is also a drug — we give it to premature newborns with underdeveloped nervous systems to bind to the adenosine receptor to stimulate the CNS in order to prevent central apnea. NeuroFAST also recognizes alcohol as addictive, and also gives a pass. Natural yeasts constantly ferment fruit while still on the vine or tree, causing it to ripen (28), yet NeuroFAST recognizes that purified alcohol is not a food. Rather, alcohol is a drug — we used to give it to women to stop premature labor before the advent of tocolytics.

Another European group with food industry ties assessed the effects of specific foodstuffs on “eating dependence” in a cohort of university students, using weight gain as the metric of food addiction. In their study, they found no difference between fats and sugars as cause for weight gain (29). However, weight gain as a metric of food addiction is inherently flawed, because some of the adolescents who manifest food addiction are of normal weight (30).

Another possible reason for the dismissal of food addiction as a psychiatric diagnosis is the phenotypic similarity to binge eating disorder (31). Many clinicians who argue against food addiction are specialists in eating disorders; however, treatment of these two entities is very different, with food addiction requiring specific food abstinence, while eating disorders do not restrict any specific foods. Therefore, differentiation of these two entities is paramount, because treatment decisions are dependent on accurate diagnosis.

In 2021, Gearhardt and Johannes Hebebrand of NeuroFAST debated the concept of food addiction in the American Journal of Clinical Nutrition (32, 33). The debate was not conclusive. Hebebrand stated, “Evidence that specific food ingredients are key determinants of addictive-like eating behavior is lacking.” (In other words, no food is specifically addictive). Gearhardt countered, stating “Highly processed foods are complex substances developed through engineering by combining reinforcing ingredients (i.e., refined carbohydrates, fat) and additives (e.g., salt) to deliver unnaturally heightened levels of reward.” (In other words, it’s not any individual compound, but rather the ultraprocessing of the specific ingredients into a new form that could lead to biochemical addiction).

## Similarities between food and drug addiction

Nora Volkow has pointed out the similarities between the neurobiology of food and drug addiction, paving the way for acceptance of this concept (34). The hedonic pathway for palatable food and drugs of abuse travels from the Ventral Tegmental Area (VTA) to the nucleus accumbens (NAc). This reward pathway is thought to have evolved to reinforce behaviors that are essential for perpetuation of the species, such as sex and feeding (35). Studies of food addiction have focused on the overlapping neural systems that may reinforce the intake of both drugs and food (36). Mesolimbic dopamine signaling between the VTA and NAc is believed to be the central feature of the hedonic pathway for both reward eating and drug abuse. Dopamine stimulation in the NAc reinforces feeding and intake of both drugs (37) and alcohol (38). The reinforcing effect of dopamine is attributed to D_2_ receptor stimulation. Indeed, dopamine signaling is believed to plays a dual role in control of feeding; inhibition of normal eating through its action in the hypothalamus, and reinforcement of pleasure eating through its action in the NAc (see mechanisms below).

## Ultraprocessed foods and addiction

If there is one food category that is most likely addictive, it would be ultraprocessed foods (UPF). The definition of UPF remains controversial. Carlos Monteiro at the University of São Paulo elaborated the NOVA system which categorizes food processing instead of nutrient content (39, 40). NOVA consists of four classes, best explained with an example (an apple): NOVA 1 (unprocessed; e.g. an apple picked off a tree); NOVA 2 (processed food ingredients; e.g. apple slices); NOVA 3 (moderately processed; e.g. apple sauce); and NOVA 4 (ultraprocessed; e.g. a fast food apple pie). Numerous investigators have epidemiologically demonstrated the association of the NOVA 4 class (41–44). However, there is still some debate as to the accuracy of the NOVA system in defining the toxicity of such foods, since some processing is either beneficial (e.g. iodized salt in bread) or non-descript (e.g. ascorbic acid as an antioxidant in sauerkraut). Furthermore, a meta-analysis of the NOVA system and all-cause mortality identifies only sugar-sweetened beverages and ultraprocessed meat products as the primary drivers of disease (45).

Nonetheless, ultraprocessing provides a rationale and mechanism, according to Gearhardt, for UPF’s to be addictive, as they are combinations of addictive ingredients that have lost their underlying fiber matrix (46). Both the combinations of food ingredients and their rapidity of absorption may yield an addictive mixture with high blood levels of ingredients are achieved (e.g. a soft drink), whereas the individual components from their native sources might not be addictive (e.g. sugar cane) (47).

## Is fast food addictive?

If there are individual substances in food that are addictive, or if the processing of food components leads to addiction, the most obvious manifestations would be within the “fast food” category. Fast food contains four components whose hedonic properties have been examined: salt, fat, caffeine, and sugar (48).

### Salt

In humans, salt intake has traditionally been conceived as a learned preference (49) rather than as an addiction. The preference for salty foods is likely learned early in life. Four- to six-month-old infants establish a salt preference based on the sodium content of breast milk, water used to mix formula, and diet (50). Because energy-dense fast foods are relatively high in salt [57], in part as a preservative to reduce depreciation, the preference for salty foods is associated with higher calorie intake. For example, a study in Korean teens showed a correlation between frequent fast food intake and preference for saltier versions of traditional foods (51). Another study examined 27 subjects undergoing opiate (mostly oxycodone) withdrawal and showed significant increases in fast food intake and weight gain over 60 days (52), suggesting “addiction transfer”. On the other hand, studies show that people can ‘reset’ their preference for less salty items. This has been demonstrated in adolescents deprived of salty pizza on their school lunch menu, and hypertensive adults who were retrained to consume a lower sodium diet over 8 to 12 weeks (49). Furthermore, at low levels, salt intake is well known to be tightly regulated. For example, patients with salt-losing congenital adrenal hyperplasia who lack the mineralocorticoid aldosterone modulate have an obligatory salt loss, which modulates their salt intake (53), until appropriate doses of fludrocortisone are supplemented. The notion that human sodium intake is “physiologically fixed” had been used to criticize recent public health efforts to reduce sodium intake so drastically (54). Nonetheless, the U.K. government engaged in a secret mass campaign to reduce public salt consumption by 30%, and saw a 40% reduction in hypertension and stroke without signs of withdrawal (55).

### Fat

The high fat content of fast food is vital to its rewarding properties. Indeed, there may be a “high-fat phenotype” among human subjects, characterized by a preference for high-fat foods and weak satiety in response to them, which acts as a risk factor for obesity (56). However, so-called “high-fat foods” preferred by people are almost always also high in carbohydrate (e.g., potato chips, pizza, or cookies). Indeed, adding sugar significantly enhances preference for high-fat foods among normal weight human subjects; yet there was no limit for preference with increasing fat content (57). Thus, the synergy of high fat along with high sugar is likely to be more effective at stimulating addictive overeating than fat alone. However, these rewarding properties of fat appear to be strictly dependent on simultaneous ingestion of carbohydrate, as low-carbohydrate high-fat (LCHF) (58) and ketogenic diets (59) consistently result in reduced caloric intake, significant weight loss, and resolution of metabolic syndrome.

Some scientists believe that dietary fat itself has addictive properties. In order to parse the differential actions, the effects of fat and sugar both separately and together (adjusting for calories) on fMRI signaling have been assessed (60). High-fat milkshakes increased brain activity in the caudate and oral somatosensory areas (postcentral gyrus, hippocampus, inferior frontal gyrus), contributing to “mouthfeel” while sugar increased activity in the insula extending into the putamen, the Rolandic operculum, and thalamus (gustatory regions), increasing “reward”. Furthermore, increasing sugar caused greater activity in those regions, but increasing fat content did not alter the amplitude. In other words, the fat increases the salience of the sugar, but it’s the sugar that effectively recruits reward circuitry.

### Caffeine

Caffeine is a “model drug” of dependence in humans (61), meeting the DSM-IV and DSM-5 criteria for tolerance, physiologic withdrawal, and psychological dependence in children (62), adolescents (63), and adults (64). Headache (64), fatigue, and impaired task performance (62) have been demonstrated during withdrawal. While adolescents and children get their caffeine from soft drinks and hot chocolate, adults get most of their caffeine from coffee and tea (65). These drinks average 239 calories and provide high amounts of sugar (66). Soft drink manufacturers identify caffeine as a flavoring agent in their beverages, but only 8% of frequent soda drinkers can detect the difference in a blinded comparison of a caffeine-containing and caffeine-free cola (67). Thus, the most likely function of the caffeine in soda to increase the salience of an already highly rewarding (high sugar) beverage. These drinks may be acting as a gateway for caffeine-dependent customers to visit a fast food restaurant and purchase fast food (68).

## The case against sugar

Finally, let’s turn to sugar. A systematic review of the literature demonstrates that ultraprocessed foods have the highest addictive potential due to their added sugar content (4). Other than caffeine, the component with the highest score on the YFAS is sugar (46). Dietary sugar is composed of two molecules in essentially equal proportion: glucose and fructose. Despite being calorically equivalent (4.1 kcal/gm), fructose and glucose are metabolized differently. In contrast to glucose, fructose does not suppress the stomach-derived hormone ghrelin (69), thus maintaining signals for hunger. A comparison of the two monosaccharides demonstrates increased risk for bingeing with fructose as opposed to glucose (70), suggesting the fructose molecule is the moiety that generates both reward and addiction responses. Through these pathways, fructose fosters overconsumption independent of energy need (71). Added sugar (and specifically the fructose moiety) activates brain reward circuitry, which in the extreme leads to addiction (48). Perhaps this is the reason that 58% of the added sugar ingested by Europeans exists within the ultraprocessed food category (72).

Although they are both ubiquitous monosaccharides, glucose and fructose are metabolized differently in the body and the brain. Glucose is the energy of life. Glucose is so important that if you don’t consume it, your liver makes it (gluconeogenesis). Conversely fructose, while an energy source, is otherwise vestigial; there is no biochemical reaction in any eukaryote that requires it. Our research has shown that when provided in excess of the liver’s capacity to metabolize fructose via the tricarboxylic acid cycle, the rest is turned into liver fat, promoting insulin resistance, and resultant NCD’s (73–75). Adding a soft drink to a fast food meal increases the sugar content 10-fold. Multivariate analysis of fast food transactions demonstrate that only soft drink intake is correlated with changes in BMI; not animal fat products (76).

### Animal studies

While the concept of human sugar addiction continues to be controversial (32, 33), the criteria for addiction are clearly met in rodents (77). Oral sucrose administration uniquely induces the acute reactant *c-fos* in the ventral tegmental area (VTA), documenting activation of the reward pathway (78). Furthermore, sucrose infusion directly into the nucleus accumbens (NAc) reduces dopamine and µ-opioid receptors similar to morphine (79), and fMRI studies demonstrate establishment of hard-wired pathways for craving (80). Sucrose administration to rodents induces behavioral alterations consistent with dependence; i.e. bingeing, withdrawal, craving, and cross-sensitization to other drugs of abuse (9). In one oft-quoted rat study, sweetness surpassed cocaine as reward (81). Recently, Minère et al. demonstrated that even in the satiated state, sucrose increases thalamic ß-endorphin and reduces α-MSH in the arcuate nucleus to drive excessive energy intake (82).

### Imaging

Human fMRI studies show that glucose and fructose have different sites of action and effects on the brain. Jonathan Purnell first explored this dichotomy by infusing each sugar intravenously, and measuring the blood oxygenation level-dependent (BOLD) signal in the brain. Glucose lit up the cortical executive control areas, but fructose suppressed the signal coming from those control areas (83). Katherine Page took this a step further by giving an oral glucose or fructose drink. She saw regional cerebral blood flow (CBF) within the hypothalamus, thalamus, insula, anterior cingulate, and striatum (appetite and reward regions) was reduced after glucose ingestion, whereas fructose ingestion reduced regional CBF in the thalamus, hippocampus, posterior cingulate cortex, fusiform, and visual cortex (84). Bettina Wölnerhanssen demonstrated lack of satiety or fullness with fructose in comparison to glucose, and fMRI lit up the limbic system (amygdala, hippocampus, orbitofrontal cortex) (85). Ania Jastreboff showed that the effects of oral fructose on dopamine activation of the nucleus accumbens was severely attenuated in obese youth (86). Consistent with other studies, fructose demonstrated lack of satiety or fullness in comparison to glucose. Lastly, the effects of fat and sugar both separately and together (adjusting for calories) on fMRI signaling have been assessed (60). High-fat milkshakes increased brain activity in the caudate and oral somatosensory areas (postcentral gyrus, hippocampus, inferior frontal gyrus); while sugar increased activity in the insula extending into the putamen, the Rolandic operculum, and thalamus (gustatory regions). Furthermore, increasing sugar caused greater activity in those regions, but increasing fat content did not alter this activation. In other words, the fat increases the salience of the sugar, but it is the sugar that effectively recruits reward and gustatory circuits. However, a recent investigation did not demonstrate a consistent relationship between the sugar in milkshakes versus brain dopamine response (87).

### Clinical

Sugar has been used for its analgesic effect in neonatal circumcision (88), suggesting a link between sugar and EOP tone. Indeed, anecdotal reports from self-identified food addicts describe sugar withdrawal as feeling “irritable”, “shaky”, “anxious” and “depressed” (18) symptoms also seen in opiate withdrawal. Other studies demonstrate the use of sugar to treat psychological dependence (89). Sugar craving can vary widely by age, menstrual cycle and time of day (90). Addiction transfer from alcohol toward sugar can be seen at any Alcoholics Anonymous meeting, where Rockstars, brownies, and Sweet-Tarts are substitutes.

### Economic

The addictive nature of sugar is even evidenced in its economics. For instance, coffee is price-inelastic, i.e. increasing price doesn’t reduce consumption much. When prices jumped in 2014 due to decreased supply, Starbuck’s sales didn’t budge an inch, due to its hedonic effects (91). As consumables go, soft drinks are the second most price inelastic, just below fast food (92). In Mexico, when the price of soda was raised by 10% by their soda tax, consumption dropped only 7.6%, indicating a distinct biochemical drive to maintain increased consumption. Similar reductions were noted after 5 years of the San Francisco Soda Tax (93).

## Two mechanisms of sugar addiction

### Indirect mechanism of addiction — inhibition of leptin signaling

Chronic fructose consumption results in hepatic *de novo* lipogenesis, which promotes fatty liver (94) and hypertriglyceridemia (95). Serum triglyceride blocks leptin’s ability to cross the blood-brain barrier (96), thus attenuating leptin’s ability to bind to leptin receptors in the VTA and extinguish mesolimbic dopamine signaling in rodents (97) and humans (A.M. 98), thus increasing reward. However, chronic dopamine stimulation down-regulates dopamine D_2_ receptors (99), thus fostering tolerance and withdrawal (100).

Although still debated, chronic hyperinsulinemia promotes leptin resistance (101). Insulin and leptin both convey information to the CNS regarding long-term peripheral energy homeostasis. Both hormones are secreted during periods of energy sufficiency, their receptors co-localize to the same VMH and VTA neurons (102), and both have similarly anorexigenic effects when administered acutely into the cerebrospinal fluid. However, chronic exposure yields a different physiologic result.

POMC neurons, exposed to a high insulin concentration *in vitro* silences their firing in response to leptin administration, resulting in leptin resistance (100). The post-receptor signal transduction pathways of the insulin receptor and leptin receptor demonstrate three separate levels of overlap, which when activated have been shown to inhibit leptin signaling insulin receptor substrate-2 (IRS-2) (103), at protein tyrosine phosphatase-1B (PTP-1B) (104), and at phosphoinositol-3-kinase (PI3K) (105). Insulin also induces Suppressor of Cytokine Signaling-3 (SOCS3) (106), which inhibits leptin signaling; conversely improved leptin sensitivity is evident in SOCS3 knockout mice (107). Thus, peripheral insulin resistance and hyperinsulinemia can lead to alterations of CNS leptin signaling centrally to foment continued weight gain (108).

Although insulin and leptin bind to separate receptors in the neurons of the VMH and VTA, they share the same signaling cascade, called insulin receptor substrate-2 (IRS2)/phosphatidyl inositol-3-kinase (PI3K) (109) and thus hyperinsulinemia may block leptin signaling. Furthermore, leptin transport across the blood brain barrier is impaired by hypertriglyceridemia, which occurs in both starvation and with the insulin resistance of obesity (96). Since leptin communicates the level of adipose stores to the brain, leptin resistance in the VMH invokes the “starvation pathway” and promotes increased caloric intake. Leptin resistance in the VTA simultaneously invokes the “hedonic pathway” and promotes increased reward of food. The majority of obese individuals manifest a state of chronic hyperinsulinemia leading to defective leptin signaling, resulting in “brain starvation” (105), which prevents the negative feedback that would normally suppress food intake (110). Thus, obesity results from chronic hyperinsulinemia, which interferes with the leptin signal, at the VMH or VTA or both (111). Thus, the insulin-leptin system paradoxically becomes a positive feedback loop or “vicious cycle” in obesity (109). Craving and appetite is accenuated, and weight accrues despite excess peripheral energy stores.

### Direct mechanism of addiction — stimulation of VTA dopamine release

The hedonic pathway that motivates the “reward” of food intake (consumption unrelated to energy need) starts in the VTA and ends in the NA. Dopamine neurotransmission from the VTA to the NA mediate the reward properties of food (112), especially under stress (113). The palatability of available food further undermines normal satiety signals and motivates energy intake independent of energy need (114). Compulsive food intake is a reflexive reaction to stimulation of this reward pathway, as evidenced by increased food intake in response to morphine microinjection into the NA (115).

Sweet foods mobilize both opioids and dopamine within the NA and establish hard-wired pathways for craving in these areas that can be identified by fMRI (80). Conversely, drugs that block D_2_ receptors (e.g. antipsychotics) are associated with a higher risk of obesity (116). In rodent models of addiction, increased addictive behavior, and pleasurable response from a food reward, as measured by dopamine release and dopamine receptor signaling, is greater after food deprivation (117).

In obese human subjects, dopamine D_2_ receptor abundance is inversely related to BMI, a sign of tolerance; and fueling a perceived need for continued food intake to provide excess stimulation of depressed circuits. In obese youth, the effects of oral fructose on dopamine activation of the NA is severely attenuated, again suggesting down-regulation of dopamine receptors (86), the neuroanatomic correlate of tolerance.

Fructose also has direct effects on increasing caloric consumption. Increasing the palatability of food by addition of sucrose undermines normal satiety signals and motivates energy intake independent of energy need (114, 118). For instance, sucrose infusion directly into the NA reduces D_2_ receptors and μ-opioid receptors similar to that of morphine (79). Both sweet and high fat foods mobilize both opioids and dopamine within the NA and establish hard-wired pathways for craving in these areas that can be identified by functional magnetic resonance imaging (80, 115). Furthermore, animal models of intermittent sugar administration, over a 3-week interval, can induce behavioral alterations consistent with dependence; i.e. bingeing, withdrawal and anxiety, craving, and cross-sensitization to other drugs of abuse (9). Neuropharmacologic analyses demonstrate reduction in D_2_ receptors in the NA, consistent with the fostering of reward and behavioral changes seen in addiction. Although anecdotal reports abound supporting human “sugar addiction”, whether this “vicious cycle” of fructose consumption is merely habituation or full-fledged dependence is not yet clear.

## Role of the food industry

Sugar is added to food either as sucrose, high-fructose corn syrup (HFCS), honey, maple syrup, or agave. In general, each are assumed to consist of half fructose, half glucose; although this percentage has recently come into question when an analysis of store-bought sodas in Los Angeles revealed a fructose content as high as 65% (119). This difference may be relevant, as fructose generates a greater reward response than does glucose. The question is whether the food industry accentuates the fructose content on purpose to promote excessive consumption. We saw similar behavior with the tobacco industry, who manipulated nicotine levels in cigarettes specifically to keep users consuming, and to convert as many as possible into “heavy users” (120). The food industry has engaged in similar practices, which has increased the percent of calories as added sugar (58%) in UPF’s. In a recent analysis, Tera Fazzino demonstrated that tobacco companies purchased food companies between 1965 and 2002 to utilize a similar strategy of increasing the sugar content of specific products to make them hyperpalatable in order to increase consumption and therefore sales (121). Interestingly, Nestlé was found to have increased the sugar content of their toddler formula in third world countries, without explanation (122). These examples argue that the food industry knows what it is doing in “spiking” their products with added sugar for their own benefit, not for the public’s. Nonetheless, the food industry turns to concepts like “nanny state” and “personal responsibility” to deflect their culpability for utilizing sugar addiction to foment sales, and in turn, the chronic disease epidemic (123).

## Reconciling the argument over food addiction *vs*. eating addiction

Systematic reviews of the literature demonstrate that ultraprocessed foods have the highest addictive potential due to their added sugar content. While sugar itself does not exhibit the DSM-IV criteria of classic tolerance and withdrawal, sugar clearly meets the DSM-5 requirements of tolerance and dependence (use despite conscious knowledge and recognition of their detriment). Yet food addiction was not codified in the DSM-5.

So how do we reconcile these two conflicting theses of “food addiction” *vs*. “eating addiction”? It would appear that of the consumables prevalent in UPF’s, sugar and caffeine possess hedonic properties. But if sugar is “food”, necessary for survival, how can it qualify as being addictive? Coca leaves are a medicinal in Bolivia, but cocaine is a drug. Opium poppies were a medicinal, but morphine is a drug. Caffeine is found in coffee (a medicinal for many), but concentrated caffeine (e.g. in weight loss remedies) is a drug. In ancient times, sugar was a spice. Through the Industrial Revolution it was a condiment. Now it’s purified, and it’s a drug. Refined sucrose is the same compound found in fruit, but the fiber has been removed, and it’s been crystallized for purity. This process of purification turned sugar from “food” into “drug” just like alcohol and caffeine (33). Like these addictive consumables, sugar is a food additive, and food additives are drugs. And it’s being added by industry to 74% of the food supply (124), because the industry knows that when they add it, we buy more.

NeuroFAST asks how foods can be addicting when they necessary to survival. Because certain foods are not necessary. We need essential nutrients that our body can’t make out of other nutrients, but there are only four classes that are truly essential: a) essential amino acids (9 out of the possible 20 found in proteins); b) essential fatty acids (such as omega-3’s and linoleic acid); c) vitamins and bioactives; and 4) minerals. None of these essential nutrients are remotely addictive. Of the hedonic substances found in food, only alcohol, caffeine, and sugar are addictive. But these are food additives, not foods.

So how do we rationalize the conflicting concepts of “food addiction” versus “eating addiction”? Because what both camps are really talking about is “food additive addiction”. They increase the salience of each other (Captain and Coke, Frappucinos) to increase consumption. When items are added to our food surpassing our ability to metabolize them, we get sick. Alcohol has always been a food additive, and caffeine dosage above 0.02% (in cola drinks) is similarly characterized as a food additive. The research shows that since there is no biochemical need for sugar, it is also a food additive, which causes addiction, excessive consumption, and NCD’s.

## Summary and the path forward

UPF’s are addictive because of the sugar (which is a food additive), while the addiction is made worse by the addition of salt and fat, which increase the salience of the sugar. Therefore, the battle over food addiction comes down to the question of “*what is food*?” Webster’s Dictionary defines “food” as “substrate that contributes to either growth or burning of an organism”. Fructose does neither, in fact it inhibits both growth and burning (125). Therefore, sugar does not meet the definition of “food”. Rather it is a food additive, just like caffeine and alcohol (we even call it “added sugar”), which are also found in food, and are also addictive. Therefore, it seems that both sides of this battle could rally around the concept of food additive addiction, of which UPF’s are emblematic (47).

Yet, I expect that this controversy will continue to rage, as long as stakeholders are not aligned, and as long as there is money involved. Using the UCSF Industry Documents Library industrydocuments.ucsf.edu, my colleagues have demonstrated that the food industry has known for years that sugar, and therefore UPF, is both toxic and addictive (126). Yet they have continued to battle in the courts and in the court of public opinion, arguing against the “Nanny State” and for personal responsibility (123). However, the science has moved us closer and closer to general acceptance. Hopefully, PET imaging studies of dopamine receptor activity in response to specific food components and linking it to systemic withdrawal can add to the mechanistic data that supports the addictive nature of UPF; although even this may not be enough for some critics. Nonetheless, the convergence of these four modalities certainly argue for UPF addiction as its own diagnostic entity, and it is our expectation that the American Psychiatric Association and the World Health Organization will soon introduce Ultraprocessed Food Addiction into the DSM-6 and ICD-11, respectively, with its own diagnostic code, so that insurance companies will reimburse treatment, and so we can lay this issue to rest and move on from this diversion with the hard work of fixing the food supply for the benefit of mankind.
